# Superior ophthalmic vein thrombosis in unique double origin: a case report

**DOI:** 10.11604/pamj.2024.47.119.42983

**Published:** 2024-03-13

**Authors:** Taha Boutaj, Hamza Lazaar, Romaissae Benkirane, Latifa Sbai, Manal Tabchi, Zineb Hilali, Rim El Hachimi, Samira Tachfouti, Abdellah Amazouzi, Ouafa Cherkaoui

**Affiliations:** 1 Ophthalmology Department “A”, Ibn Sina University Hospital (*Hôpital des Spécialités*), Mohammed V University, Rabat, Morocco

**Keywords:** Diabetes, exophthalmos, infection, magnetic resonance imaging, case report

## Abstract

Superior ophthalmic vein thrombosis (SOVT) is a rare orbital pathology. It can cause serious complications if it isn´t diagnosed appropriately. It can be secondary to many etiologies, septic or aseptic ones. Diabetic ketoacidosis (DKA) may disturb the vascular endothelium and promote a prothrombotic state. The presence of which is related to a significantly increased risk of morbidity and mortality. We report the case of a 45-year-old woman who presented a SOVT revealing DKA. Orbit magnetic resonance imaging (MRI) showed thrombosis of the right superior ophthalmic vein. A treatment based on thrombolytic treatment, associated with antibiotic coverage and a glycemic balance was initiated. This case highlights the importance of considering both infection and diabetes as an important part of the diagnosis and management of SOVT.

## Introduction

Superior ophthalmic vein thrombosis (SOVT) is a rare orbital pathology [1]. It can cause serious complications if it isn´t diagnosed appropriately [2]. It can be secondary to many etiologies, septic or aseptic ones. DKA may disturb the vascular endothelium and promote a prothrombotic state. The presence of which is related to a significantly increased risk of morbidity and mortality. We report the case of a 45-year-old woman who presented a SOVT revealing diabetic ketoacidosis. Orbit MRI showed thrombosis of the right superior ophthalmic vein. A treatment based on thrombolytic treatment, associated with antibiotic coverage and a glycemic balance was initiated. This case highlights the importance of considering both infection and diabetes as an important part of the diagnosis and management of SOVT.

## Patient and observation

**Patient information:** we report the case of a Moroccan 45-year-old patient with maternal hx of diabetes mellitus, who arrived at the ophthalmology emergency presenting abrupt onset of right exophthalmos and ptosis.

**Figure 1:**
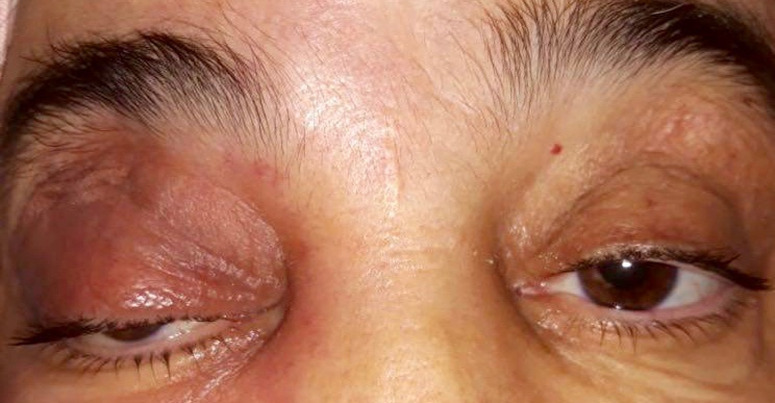
clinical photograph of the patient at emergency room showing exophthalmos with ptosis and right eyelid inflammation (right eye)

**Clinical findings:** on the ophthalmologic examination, visual acuity was 20/40 in both eyes. The pupillary light reflex was present in both eyes. Intraocular pressure was normal. Slit-lamp biomicroscopic examination showed a painful and pulsating right exophthalmos, associated with ptosis and right eyelid inflammation (Figure 1). Ocular motility was limited upwards in the right eye. The anterior segment was normal. After dilatation, the funduscopy of both eyes was normal, without any element of venous congestion.

**Figure 2:**
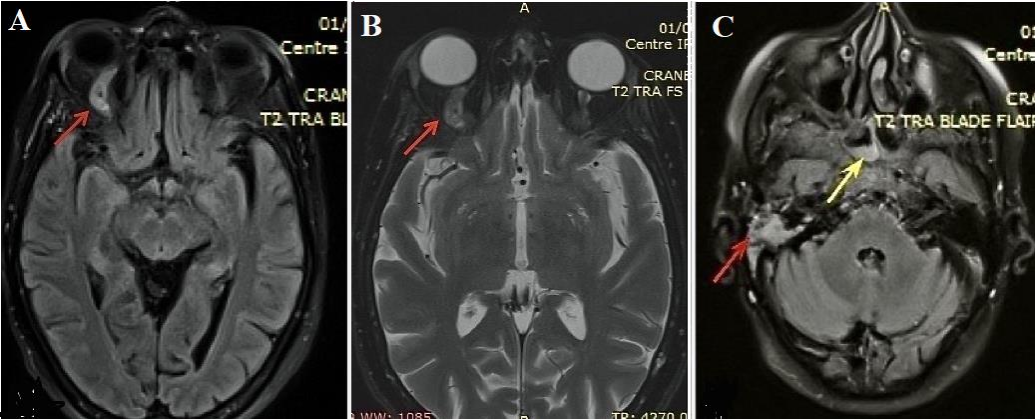
orbito-cerebral angio magnetic resonance imaging; axial images: (A,B) thrombosis of the right superior ophthalmic vein, with inflammatory infiltrate in the upper right rectus muscle; (C) sphenoidal sinusitis, and right otitis media

**Diagnostic assessment:** an emergency standard biological examination was performed: COVID-19 reverse transcription polymerase chain reaction (RT-PCR) was negative. The patient's white blood cell (WBC) count was 12.000/mm^3^. C-reactive protein (CRP) was at 72 mg/l. Blood glucose was at 35 mmol/l, and the urine ketone test was at 15 mmol/l (3+). After stabilization of the patient in the intensive care unit, an orbit MRI was performed urgently, showing thrombosis of the right superior ophthalmic vein, an inflammatory infiltrate in the upper right rectus muscle, a filling of the sphenoid sinus with a hydro-aeric level in the reported with acute sphenoidal sinusitis, and an acute right otitis media (Figure 2). The diagnosis of SOVT revealing DKA was retained.

**Therapeutic intervention:** after a multidisciplinary team decision, it was decided to initiate a thrombolytic treatment, associated with antibiotic coverage and a glycemic balance.

**Follow-up and outcomes:** after two weeks of hospitalization, ptosis improved with the disappearance of exophthalmos. She has preserved the range of movement in her eyes. Follow-up after 1 month was uneventful.

**Patient´s perspective:** the patient shared their perspective on the treatment with a disappearance of the symptoms. She could return to her normal activity.

**Patient consent:** the patient gave informed consent.

## Discussion

The superior ophthalmic vein is the main draining venous element in the orbit. Superior ophthalmic vein thrombosis (SOVT) is a very rare orbital and palpebral pathology [1]. It can cause serious complications if it is not detected and managed promptly and effectively [2]. Congestion of the superior ophthalmic vein is secondary to many causes: aseptic and septic ones [3]. The aseptic ones are facial trauma, orbit neoplasia, Tolosa-Hunt syndrome, inflammation, and hypercoagulability state: lupus, antiphospholipid syndrome, sarcoidosis, Behcet, pregnancy, and DKA. This DKA may disturb the vascular endothelium and promote a prothrombotic state [4]. Studies have shown that free protein S and protein C activity levels decrease and von Willebrand factor levels increase during DKA [5].

Aseptic causes are mostly non-aggressive. Their management depends essentially on anticoagulation. The septic causes on the other side are more aggressive: orbital cellulitis, sinusitis, and cavernous sinus thrombosis [6]. Orbital infections by aerobic or anaerobic organisms are the most common cause of the SOVT. The most common pathogens causing orbital cellulitis are *Staphylococcus aureus* and *Streptococcus* family [7]. COVID-19 was also implicated in the pathogenesis of SOVT: COVID-19 is thought to induce a systemic inflammatory response, endothelial dysfunction, and a hypercoagulative state which predisposes patients to form systemic thrombi [8]. When the cause is septic, the clinical presentation is more aggressive, and the treatment includes anticoagulation and antibiotics. About the clinical presentation, SOVT can be unilateral or bilateral. Symptoms are caused by impaired orbital venous drainage. It includes pain, chemosis, eyelid edema, proptosis, limited ocular motility, orbital swelling, and impaired visual acuity.

Due to the difficulty of clinical and etiological diagnosis, neuroimaging is capital in the diagnosis of SOVT. It can be detected on post-contrast computed tomography or magnetic resonance imaging [9]. Delayed management can be exposed to severe complications. Superior ophthalmic vein thrombosis (SOVT) may progress to cavernous sinus thrombosis with involvement of the cranial nerves within the cavernous sinus and intracranial complications [3]. The treatment of SOVT depends on the underlying etiology. It includes anticoagulation, antibiotics, and surgery. There is no consensus or guidelines addressing its management. The aseptic SOVTs required only anticoagulation. However, the septic ones are more aggressive and need both anticoagulations and antibiotics. Superior ophthalmic vein thrombosis (SOVT) with orbital abscess required surgical drainage [3]. In our case, the septic cause (otitis and sinusitis) and aseptic (DKA) were both responsible for the SOVT in variable proportions, but the clinical improvement under anticoagulants and antibiotics are more suggestive of an infectious cause potentiated by unbalanced diabetes.

## Conclusion

Superior ophthalmic vein thrombosis is a rare orbital pathology. Early diagnosis and treatment are important to avoid complications. Treatment is etiological.
